# Recovery from impaired muscle growth arises from prolonged postnatal accretion of myonuclei in *Atrx* mutant mice

**DOI:** 10.1371/journal.pone.0186989

**Published:** 2017-11-02

**Authors:** Michael S. Huh, Kevin G. Young, Keqin Yan, Tina Price-O’Dea, David J. Picketts

**Affiliations:** 1 Regenerative Medicine Program, Ottawa Hospital Research Institute, 501 Smyth Road, Ottawa, ON Canada; 2 Department of Biochemistry, Microbiology & Immunology, University of Ottawa, Ottawa, ON Canada; 3 Department of Cellular and Molecular Medicine, University of Ottawa, Ottawa, ON Canada; Universitat Wien, AUSTRIA

## Abstract

Reduced muscle mass due to pathological development can occur through several mechanisms, including the loss or reduced proliferation of muscle stem cells. Muscle-specific ablation of the α-thalassemia mental retardation syndrome mutant protein, Atrx, in transgenic mice results in animals with a severely reduced muscle mass at three weeks of age; yet this muscle mass reduction resolves by adult age. Here, we explore the cellular mechanism underlying this effect. Analysis of *Atrx* mutant mice included testing for grip strength and rotorod performance. Muscle fiber length, fiber volume and numbers of myofiber-associated nuclei were determined from individual EDL or soleus myofibers isolated at three, five, or eight weeks. Myofibers from three week old *Atrx* mutant mice are smaller with fewer myofiber-associated nuclei and reduced volume compared to control animals, despite similar fiber numbers. Nonetheless, the grip strength of *Atrx* mutant mice was comparable to control mice when adjusted for body weight. Myofiber volume remained smaller at five weeks, becoming comparable to controls by 8 weeks of age. Concomitantly, increased numbers of myofiber-associated nuclei and Ki67^+^ myoblasts indicated that the recovery of muscle mass likely arises from the prolonged accretion of new myonuclei. This suggests that under disease conditions the muscle satellite stem cell niche can remain in a prolonged active state, allowing for the addition of a minimum number of myonuclei required to achieve a normal muscle size.

## Introduction

The postnatal growth of skeletal muscle encountering experimental overload hypertrophy or following exercise requires the contribution of new myoblast nuclei to the muscle fiber syncytium to allow for an increase in fiber size [1–3]. The production of myoblasts, derived from the muscle satellite cell niche, requires the coordinated expression of multiple genes which restrict the lineage potential of the cells and initiate the expression of muscle-specific proteins. Chromatin remodeling proteins facilitate the ability of muscle determining transcription factors, including Myf5 and MyoD, to access their chromosomal binding sites [4]. Our previous work has demonstrated an important role for the Atrx chromatin remodeling protein in postnatal myoblast production [5].

In humans, mutant ATRX protein underlies the α-thalassemia mental retardation syndrome, characterized by severe intellectual disability, urogenital abnormalities, a characteristic facial appearance, and, in many instances, alpha-thalassemia [6]. In addition, many patients display hypotonia at birth, have delayed ambulation and kyphosis. While these features of the disease are often attributed to a CNS defect, our characterization of muscle-specific *Atrx* conditional knockout (cKO) mice has indicated that these muscle deficiencies may result from a combination of CNS and skeletal muscle defects [5].

Atrx interacts in a complex with Daxx to load the variant histone H3.3 onto chromatin in a replication-independent manner [7–10]. Atrx binds to repetitive sequences, many of which are G-rich and form DNA secondary structures called G-quadruplexes (G4) [11]. Of note, telomere repeats show a high propensity to form G4 structures and cells lacking Atrx demonstrate proliferation defects and increased cell death resulting from replication stress and telomere fragility [5, 12, 13]. Indeed, the hypoproliferation of Atrx-deficient cells results in reduced tissue size. In this regard, muscle-specific *Atrx* cKO mice, generated by breeding *Atrx*^*f/f*^ mice to a *Myf5-Cre* driver line, had a deficiency in myoblast expansion resulting in animals with severely reduced muscle mass at three weeks of age [5]. Despite their small size, these animals had normal numbers of resident satellite cells that were capable of activation and initiating cell division. Surprisingly, the size difference was no longer present in adult animals, although the regenerative capacity of satellite cells remained impaired, as suggested by defective muscle repair following cardiotoxin injury [5].

In mice, the accretion of myonuclei from the fusion of proliferating satellite cell-derived myoblasts onto the myofiber occurs primarily within the first three weeks following birth. In the adult, muscle mass is primarily generated from hypertrophy of the existing muscle fibers with little addition of new myoblast nuclei, while the accretion of new myonuclei occurs primarily during regeneration following acute muscle injury or stress from overloads [14]. The *Atrx* cKO mice therefore provide us with a model to assess the mechanisms underlying muscle growth in animals with impaired myoblast proliferation and developmentally delayed growth.

## Materials and methods

### Mouse husbandry

*Myf5-Cre* mice were obtained from P. Soriano and maintained on a 129/Sv background. *Atrx* exon 18 floxed mice were maintained on a C57BL/6 background as previously described [5]. Animals were housed in the University of Ottawa Animal Care and Veterinary Service facility, and monitored daily. The mice described in this study are not uniquely prone to illness or distress; illness with any mice in the facility is dealt with according to the University of Ottawa ACVS policies (http://research.uottawa.ca/acvs/policies-procedures). All experiments involved either the use of awake animals, or the collection of tissues following euthanasia with CO_2_. Mice used for analysis were all male. All animal experiments were approved by the University of Ottawa's Animal Care ethics committee, with the guidelines set out by the Canadian Council on Animal Care. In addition, we have included the ARRIVE guidelines checklist developed for reporting *in vivo* animal research experiments (S1 Fig).

### Muscle fiber morphometric analysis

Live myofibers were dissociated and cultured from the extensor digitorum longus muscle and soleus muscles as described previously [5]. Freshly isolated fibers were fixed in 2% PFA, stained with DAPI and mounted onto slides. Images were taken on Axio Imager M1 (Zeiss) and morphometric analysis was performed using ImageJ software suite.

### Grip strength and rotorod analysis

Mouse grip strength was measured on a DF II series digital force gauge (AMETEK Measurements & Calibration Technologies) and mouse neuromuscular function such as balance, coordination, and endurance was assessed by rotorod latency tests (IITC Inc. Life Science). Grip strength and rotorod tests were conducted at the University of Ottawa Behavioral Core.

### Ki67 quantification

Paraffin embedding and Ki67 immunohistochemistry were performed at the University of Ottawa pathology laboratory. Ki67 positive myofiber-associated nuclei were quantified from serial cross sections prepared from the mid-belly of paraformaldehyde-fixed EDL and soleus muscles. Images were acquired on Axio Imager M1 (Zeiss) and analyzed with Adobe Photoshop.

### Immunofluorescent labeling

Muscle cross sections, taken from the mid-belly of each muscle type, were cut from mice which had been perfused with 4% paraformaldehyde. The muscle was further immersed in 4% paraformaldehyde overnight at 4°C, washed in PBS, then immersed in 30% sucrose at 4°C until saturated. OCT embedded muscle was frozen using liquid nitrogen, and stored at -80°C. 12 μm cryostat sections were used for immunolabeling. A standard antigen retrieval protocol using a pH 6.0 sodium citrate solution, and heating in a microwave, was performed prior to labeling. Sections were labeled with antibodies against Ki67 (rabbit pAb; Abcam; or rat mAb, clone 11F6, Biolegend), Pax 7 (mouse mAb, clone Pax7, R&D Systems), MyoD (mouse mAb, clone 5.8a, Dako), or Atrx (rabbit pAb, H-300, Santa Cruz). An Alexa-fluor 488 conjugated isotype-specific secondary (anti-mouse IgG1, Thermo Scientific) was used to detect the Pax7 and MyoD antibodies; appropriate anti-rat and anti-rabbit secondary antibodies conjugated to Alexa-fluor 555 or Alexa-fluor 647 were used to detect the other primary antibodies. Wheat germ agglutinin (Thermo Fisher Scientific) was used to label the myofiber extracellular matrix. For fiber type analysis, mouse IgM monoclonal antibody supernatants targeting myosin heavy chain (MHC) type IIb (clones BF-F3) and MHC type IIa + I (BF-32) (Developmental Studies Hybridoma Bank) were used in combination with a rabbit anti-laminin pAb (Sigma). The same anti-IgM-Cy3 antibody was used to detect both anti-MHC antibodies. Conventional wide-field fluorescence microscopy was used for imaging the sections with 20x (0.8NA) or 40X (1.3NA) objective lenses. Acquisition and post-processing was performed with Axiovision.

### SYBR green qPCR analysis

RNA was harvested from liquid nitrogen freeze fractured EDL and soleus muscles using Trizol (Thermo Fisher Scientific) and reverse transcribed with SuperScriptIII (Thermo Fisher Scientific). cDNA was used for expression analysis with oligonucleotide primers (Sigma) specific for *myostatin*, *Acvr2A*, *InhbA*, *IL-6*, *TNF*, myomaker, and follistatin. Oligonucleotide primers for GAPDH were used to amplify a reference cDNA. qPCR was performed on a Stratagene Mx3000P system using a SensiFAST SYBR Lo-ROX kit (Bioline). Relative expression fold-changes were calculated using the 2^-ΔΔCt^ method, and range was calculated using the standard deviation of the Ct values.

## Results

### *Atrx* cKO mice have reduced muscle mass but equivalent grip strength

To further our understanding of the role of Atrx in tissue development, two mouse models have been generated; a conditional allele that results in protein ablation when bred to specific Cre driver lines and a hypomorphic mutation that mimics a human mutation in exon 2 producing reduced levels of an N-terminally truncated protein [15, 16]. Although human patients are primarily hypomorphs, the Atrx-null model has shown similar, albeit slightly more severe, neuronal and retinal phenotypes than mice with the hypomorphic mutation [16, 17], thereby validating the cKO mice as a good model to interrogate Atrx function. In this study, we utilized the conditional *Atrx* allele bred to a *Myf5*-Cre driver line to generate muscle-specific *Atrx* cKO mice.

**Fig 1:**
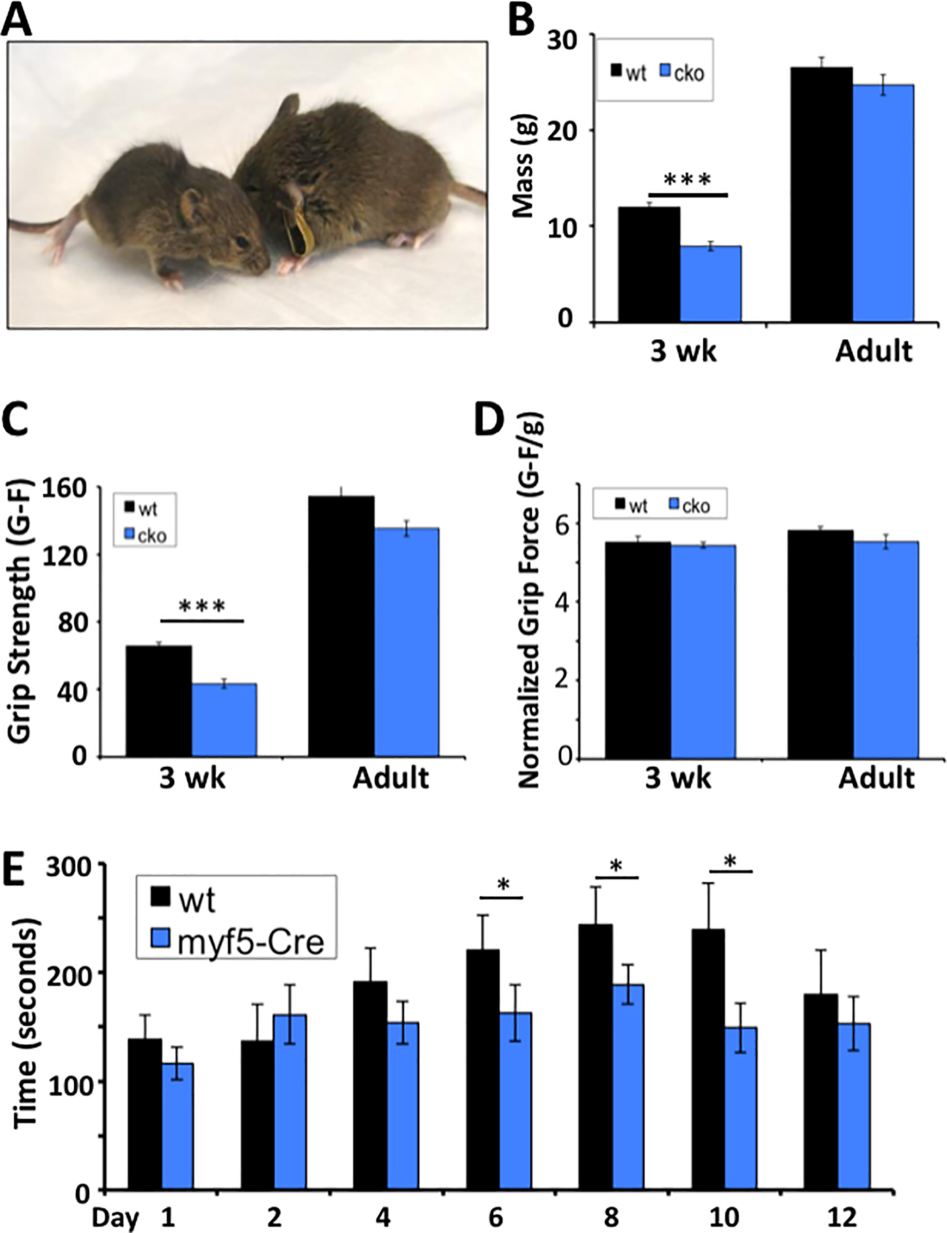
Characterization of Atrx cKO mice. (A, B) Body mass of *Atrx* cKO mice compared to littermate controls at 3 weeks (n = 11, 9; WT, cKO) and 8–9 weeks (n = 8, 11; WT, cKO) of age (mean ± SEM; ***p<0.001 by student’s t-test) (C) Grip strength analysis of *Atrx* cKO mice vs. littermate controls at 3 weeks and 8–9 weeks (adult) of age. (mean ± SEM; ***p<0.001 by student’s t-test) (D) Grip strength of *Atrx* cKO mice normalized to body weight. (mean ± SEM) (E) Rotorod latency time of *Atrx* cKO mice and littermate controls. Animals were subjected to the roto-rod apparatus for two consecutive days, and then every two days afterwards until day 12. Five trials were timed and recorded for each mouse at each experimental time point. (n = 8; mean ± 95% CI; *p<0.05 by ANOVA repeated measures statistical analysis).

We first examined body mass in young (three week-old) and adult (eight to nine week-old) mice lacking the production of Atrx in myoblast cells to quantitate differences with wild type littermates. We observed a significant reduction in body mass in juvenile *Atrx* cKO mice compared to controls at three weeks of age (*Atrx* cKO 7.92g ± 0.45g vs. WT 11.95g ± 0.47g) but this difference resolved by the time the animals reached adulthood (*Atrx* cKO 24.71g ± 1.05g vs. WT 26.51g ± 1.09g; Fig 1A and 1B). At an intermediate time of five weeks, where a body weight difference persists (*Atrx* cKO 21.6g ± 0.55g vs. WT 24.40 ± 0.38g), *Atrx* cKO hindlimb muscles are smaller, though only marginally so when adjusted for body weight (S2 Fig). Gross muscle morphology and fiber composition was similar between *Atrx* cKO and WT mice at this time point (S3 Fig).

We next asked whether the function of the muscle was compromised in the mutants. In this regard, muscle grip strength was assessed at three weeks of age when the animals were smaller in size and in adults when muscle and body mass was equivalent. We observed a reduction in grip strength at three weeks but not in the adult animals (Fig 1C). However, when the grip strength was normalized to body weight there were no differences (Fig 1D), suggesting that muscle strength was equivalent to their control littermates. We conclude from these experiments that the young *Atrx* cKO mice have reduced muscle mass but maintain grip strength. While this is suggestive of normal muscle function a larger battery of tests to fully assess muscle function is required.

### Muscle recovery following exercise is compromised in *Atrx* cKO mice

Adult *Atrx* cKO mice showed poor recovery from muscle injury following cardiotoxin injection indicating that muscle regeneration was compromised [5]. Cardiotoxin injection induces severe damage to the muscle requiring rampant expansion of the satellite cells to repair the injury. While this identified a genomic stability role for Atrx during satellite cell replication, Atrx-null cells could manage the damage if the replicative phase was not prolonged [5]. In this regard, we wanted to assess whether the animals could cope with minor muscle injury, such as daily exercise. As such, we subjected the animals to the roto-rod apparatus and measured the time to fall (latency time). Over the first four days the mutant mice performed as well as the control mice (Fig 1E). However, by day six they fell off the rod much sooner than the wild type mice and this pattern was maintained until day twelve, by which time the control animals were also showing fatigue in their ability to stay on the apparatus (Fig 1E). These studies demonstrate that despite an increase in muscle mass and a normal grip strength compared to wild type mice, the muscle from adult *Atrx* cKO animals still exhibits poor recovery after exercise.

### Prolonged myoblast proliferation occurs in *Atrx* mutant muscle

Given that the *Atrx* cKO mice are defective in myoblast proliferation yet have normal muscle mass as adults, we next asked whether the increase was the result of hypertrophy, and whether they continued to accrue new myofiber-associated nuclei beyond the three week window. We isolated mRNA from WT and *Atrx* cKO soleus and EDL muscle at five weeks to explore whether there was decreased expression of the negative regulators of muscle hypertrophy, namely *myostatin*, *Inhibin A*, or their receptor *Acvr2A*. We observed no significant differences in the expression of these transcripts in *Atrx* cKO muscle (S4 Fig). We further assessed the expression of IL-6, myomaker, and follistatin, each of which facilitates muscle hypertrophy [18–20], and TNF, which is associated with both the promotion of myoblast proliferation [21] and with muscle wasting [22]. No major differences existed in the expression of any of these transcripts in the *Atrx* cKO muscle, though there was a small, statistically significant increase in the level of follistatin transcript in the soleus muscle. These data did not indicate the presence of hypertrophic growth consistent with that seen in models involving muscle workload-stimulated hypertrophy.

**Fig 2:**
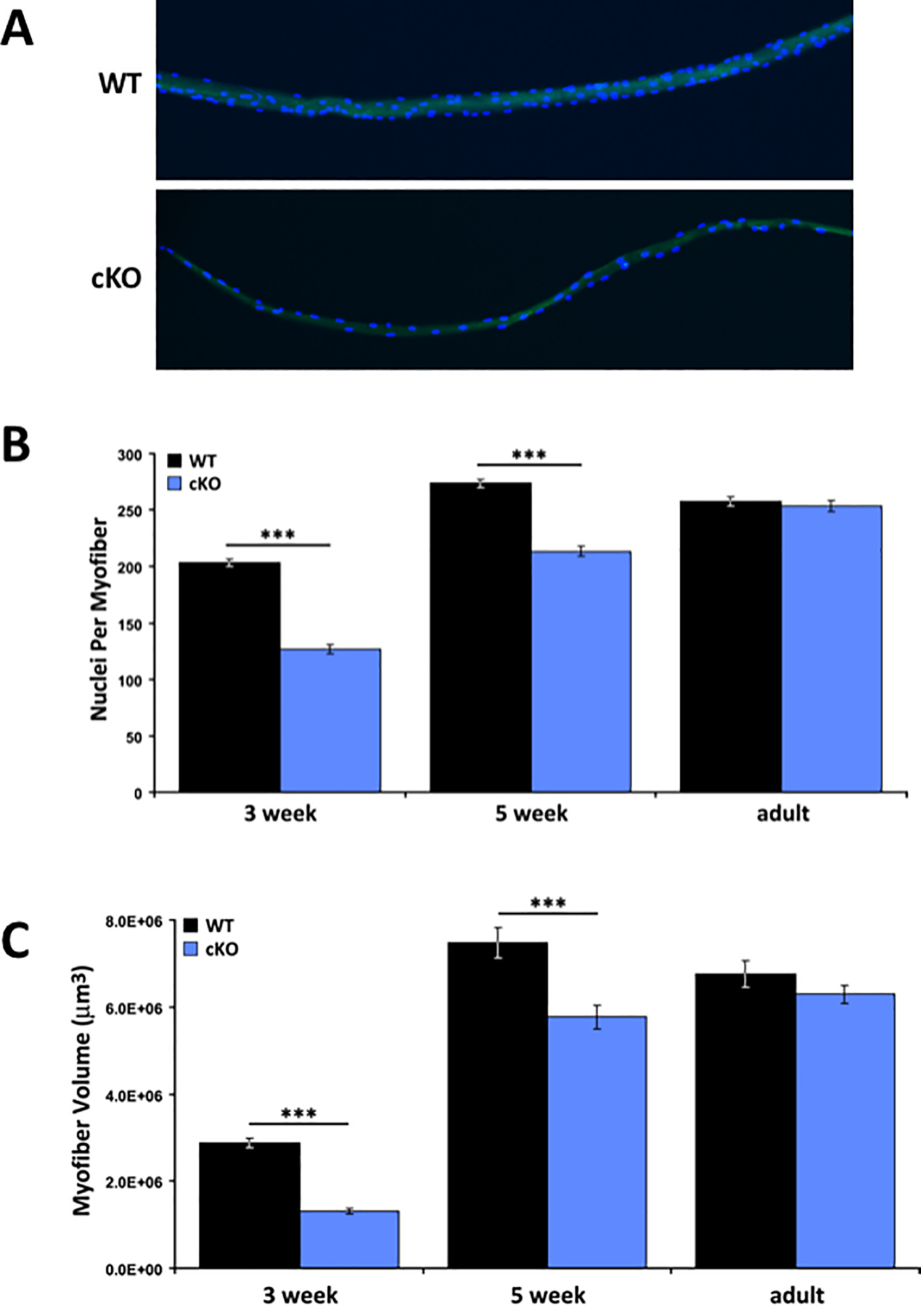
Myofibers from *Atrx* cKO mice are smaller and contain fewer nuclei. (A) DAPI stained nuclei on representative fibers isolated from 3-week old WT and *Atrx* cKO EDL muscle. The green is autofluorescence, used to visualize the fibers. (B) Average myonuclear content per fiber in EDL muscle in *Atrx* cKO and littermate controls (mean ± SEM; ***p<0.001 by student’s t-test; 3 week WT, cKO: n = 110, 119 fibers; 5 week WT, cKO: n = 160, 149 fibers; adult WT, cKO: n = 183, 212 fibers). (C) Average volume per fiber in EDL muscle in *Atrx* cKO and littermate controls. (mean ± SEM; ***p<0.001 by student’s t-test; 3 week WT, cKO: n = 111, 122 fibers; 5 week WT, cKO: n = 170, 178 fibers; adult WT, cKO: n = 186, 214 fibers). Fibers were collected from 2 mice/group at 3 weeks; 3 mice/group at 5 weeks and adult ages.

To determine the basis of the increase in muscle mass between young and adult *Atrx* cKO animals, we isolated individual myofibers from the EDL muscle of young (three weeks), juvenile (five weeks) and adult (eight to nine weeks) *Atrx* cKO and wild type mice for morphological analyses. First, we examined individual fibers for the number of myofiber-associated nuclei (Fig 2A and 2B). In wild type mice, the average number of nuclei per fiber increased from three weeks (203.31 ± 3.42; n = 100 fibers) to five weeks (273.47 ± 3.55; m = 160 fibers). The 35% increase in accretion of nuclei between three to five weeks likely stems from the fusion of differentiating myoblasts coinciding with the end of the postnatal growth phase. Indeed, the number of nuclei remained fairly constant in the adult animals (257.58 ± 3.88; n = 183 fibers). By comparison, the *Atrx* cKO muscle fibers had 40% fewer nuclei at three weeks (126.82 ± 4.23; n = 119 fibers; p<0.001, t-test), but demonstrated a 70% increase in this number by five weeks (213.52 ±4.55; n = 149 fibers; p<0.001, t-test). This suggests that there was greater myonuclei accretion during this two week period when the growth phase in control mice was slowing down. Certainly, this is reflected in the adult numbers (253.28 ± 4.86; n = 212 fibers) where a further 20% increase from five week-old mice was observed, resulting in equivalent numbers to the adult control mice.

**Fig 3:**
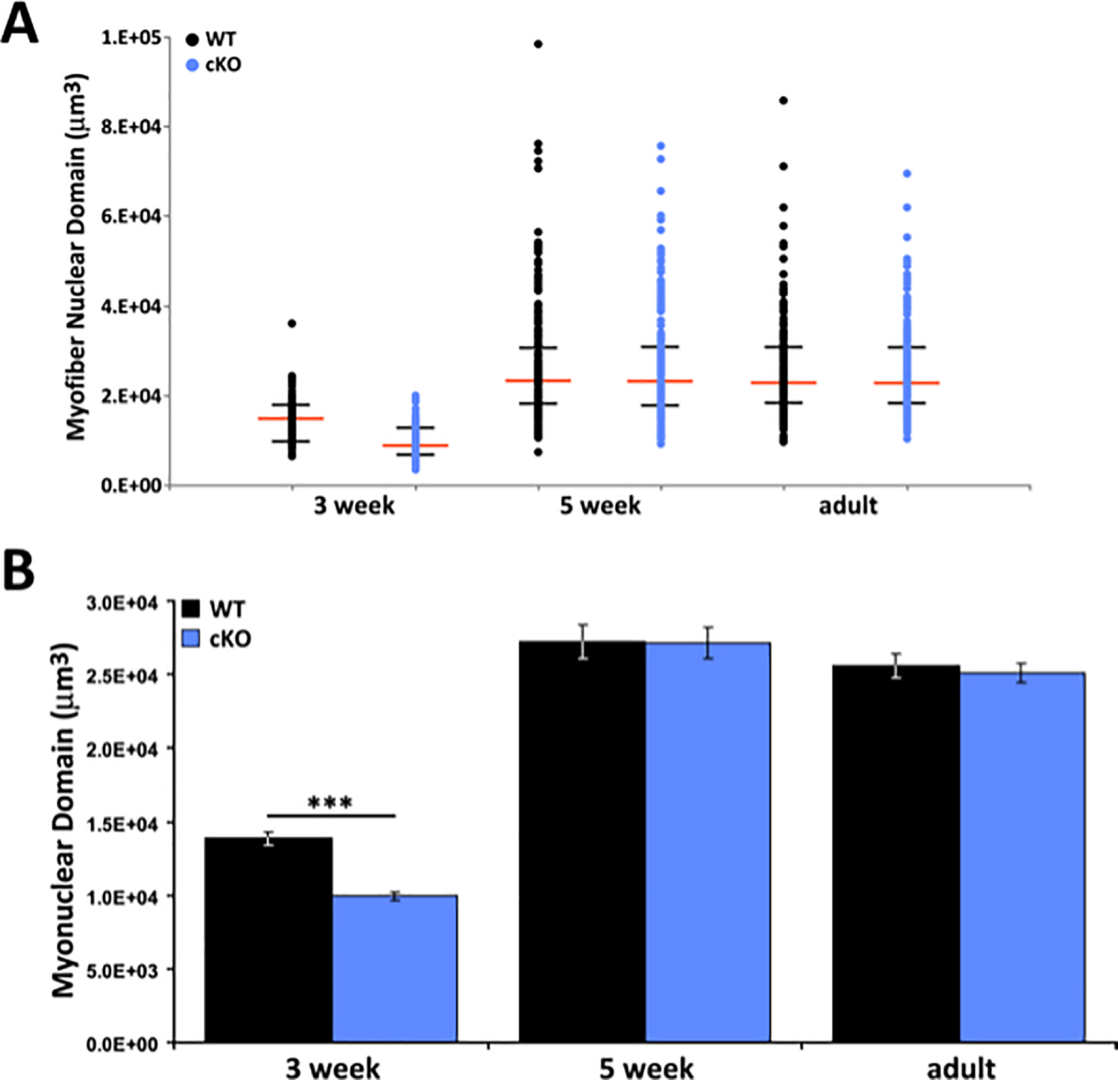
Recovery of myonuclear domain size by 5 weeks of age. (A) Distribution of individual myofibers from the EDL muscle as plotted by myonuclear domain (volume/nuclei) per fiber in *Atrx* cKO and littermate controls. Median and quartile points are represented for each animal group by a red and black hash marks respectively. (3 week WT, cKO: n = 110, 119 fibers; 5 week WT, cKO: n = 160, 149 fibers; adult WT, cKO: n = 183, 212 fibers). (B) Average myonuclear domain per fiber in EDL muscle in *Atrx* cKO and littermate controls (mean ± SEM; ***p<0.001 by student’s t-test). Fibers were collected from 2 mice/group at 3 weeks; 3 mice/group at 5 weeks and adult ages.

Similarly, we observed significantly reduced fiber volume (Fig 2C) and length (S5 Fig) at both three and five weeks of age in the mutant animals compared to control littermates. In adult mice, these differences had also dissipated. Since myoblasts fuse onto existing fibers and thereby contribute their cytoplasm and nucleus, it was not surprising that these metrics were also altered. However, when we normalized the data by calculating the myonuclear domain (volume/nuclei) of individual fibers, we observed a significant difference at three weeks (WT: 13857 ± 436 μm^3^/nuclei, n = 110; cKO: 9953 ± 300 μm^3^/nuclei, n = 119; p<0.001, t-test) but not at five weeks (WT: 27231 ± 1134 μm^3^/nuclei, n = 160; cKO: 27147 ± 1088 μm^3^/nuclei, n = 149) or adult (WT: 25612 ± 812 μm^3^/nuclei, n = 183; cKO: 25065 ± 664 μm^3^/nuclei, n = 212) (Fig 3). Taken together, these studies suggest that there is a two-fold increase in myonuclei accretion between three and five week old mutant animals compared to WT mice, and that this is the major effector restoring muscle mass.

**Fig 4:**
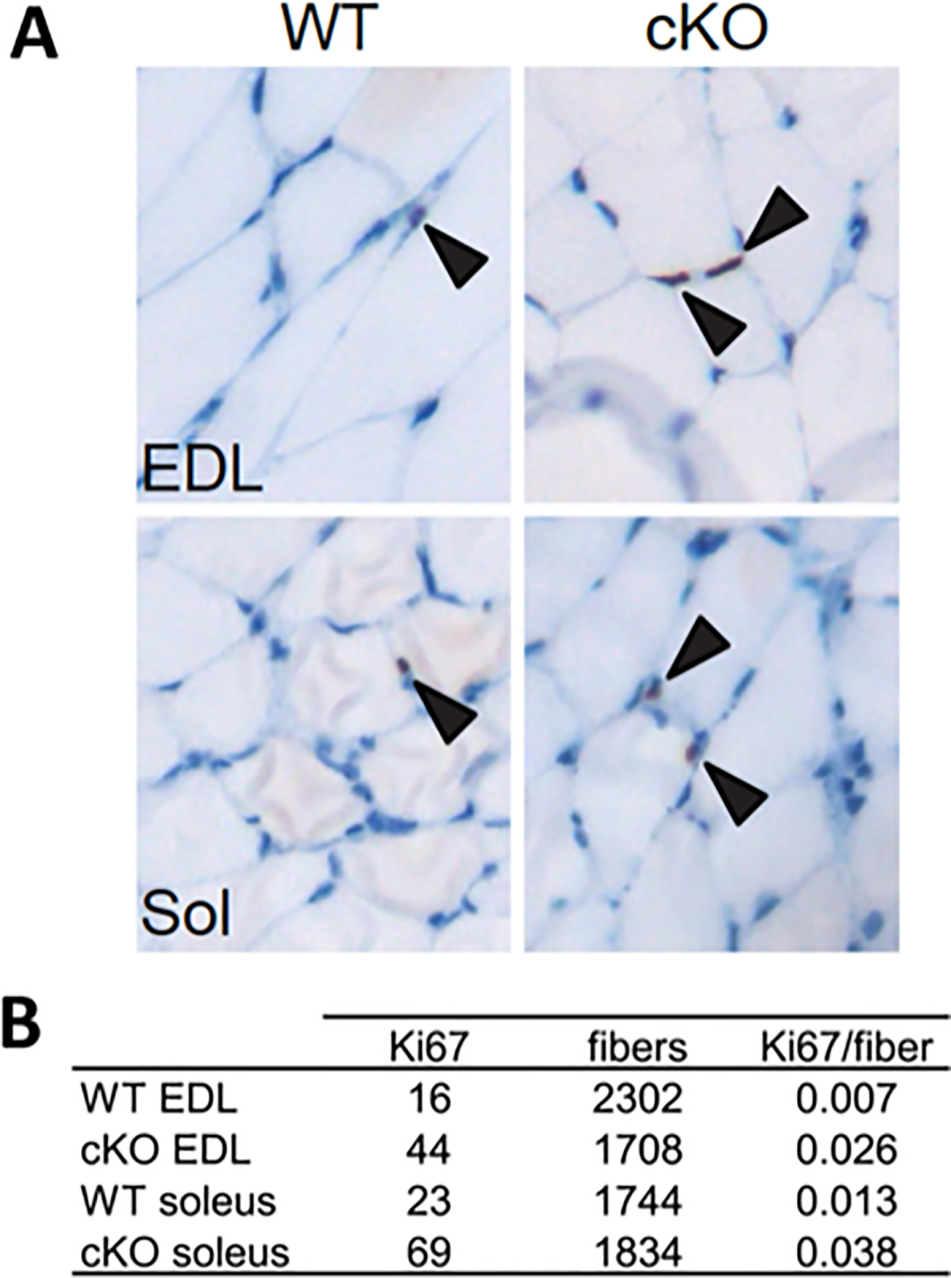
*Atrx* cKO muscle contains increased numbers of proliferating cells. (A) Representative cross-sectional images of 5-week old EDL or Soleus (Sol) muscle from WT and *Atrx* cKO mice stained with anti-Ki67 antibody. Arrowheads indicate Ki67^+^ myofiber-associated nuclei. (B) Quantification of Ki67^+^ nuclei in 5-week old EDL and Soleus muscles indicated an approximately 3-fold increase in the mutant muscle. n = 3 mice/group.

Such a dramatic increase in myonuclei accretion implies that there is an increased number of proliferating myoblasts that ultimately differentiate and fuse to the fiber during this time frame. To assess whether there might be an increased number of proliferating myoblasts within the *Atrx* cKO muscle, we first sectioned EDL and soleus muscles and stained them with Ki67, a marker of active proliferation. This preliminary experiment demonstrated that Ki67^+^ nuclei were present in all sections regardless of Atrx status (Fig 4A). To quantify any change in proliferating myoblasts, we examined >1500 myofibers from cross sections of EDL and soleus muscle from five-week old WT and *Atrx* cKO mice and scored the proliferation status of associated myoblasts after staining with Ki67. In the EDL muscle from WT mice 0.7% of fiber-associated nuclei were Ki67^+^, compared to 2.6% in *Atrx* cKO EDL, indicating a 3.7-fold increase in proliferation in the mutant mice (Fig 4B). Similarly, we observed a 2.9-fold increase in proliferating cells in the soleus muscle of the *Atrx* cKO mice compared to control littermates (Fig 4B).

**Fig 5:**
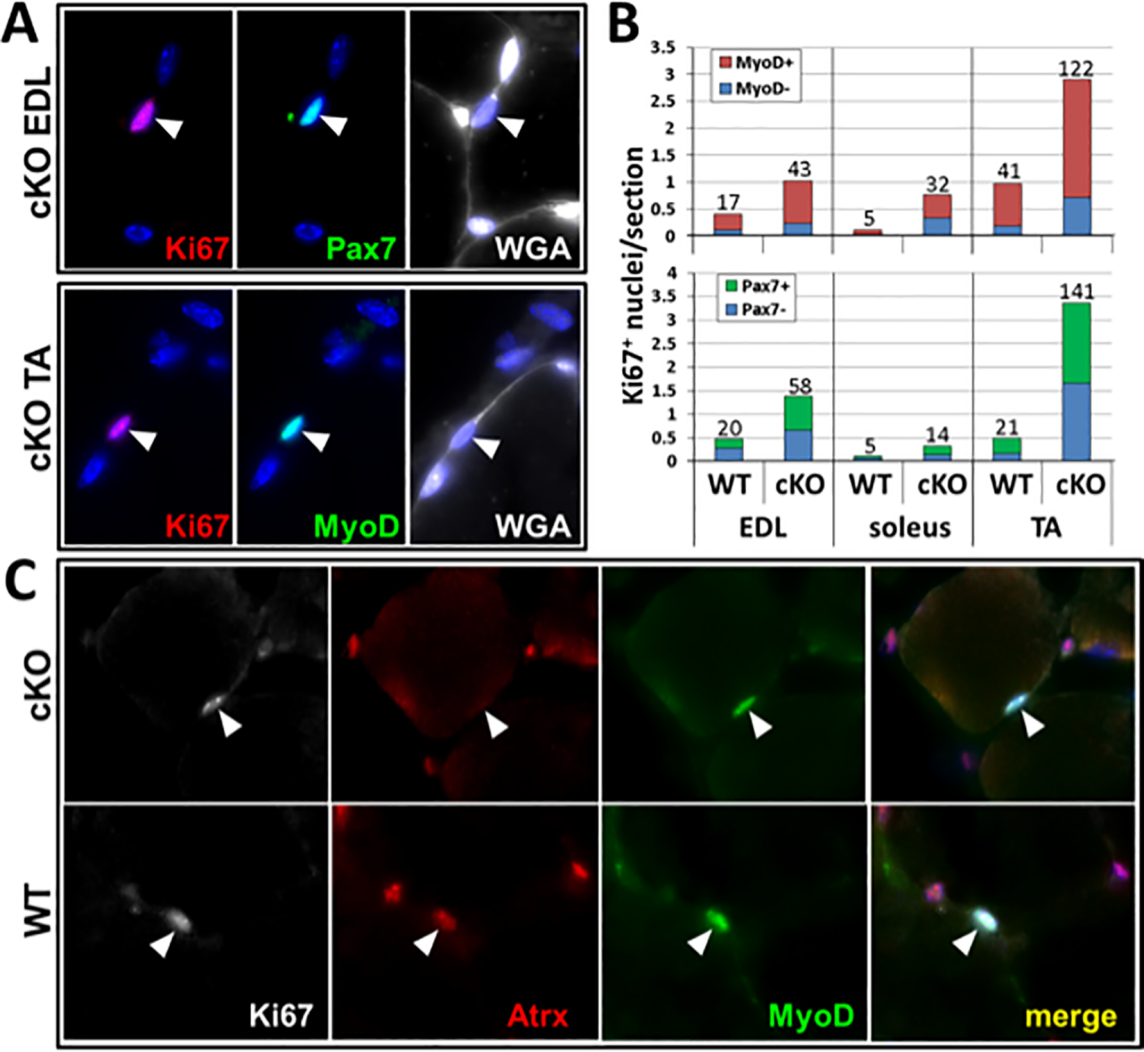
Proliferating cells are primarily MyoD- and Pax7-positive myoblasts. (A, B) The majority of Ki67^+^ cells in *Atrx* cKO and wild type muscle co-express the myoblast markers MyoD and Pax7. (A) Arrowheads indicate nuclei labeled for Ki67 and either MyoD or Pax7. Hoechst-stained nuclei are shown in blue. Myofiber-associated Ki67^+^ nuclei were counted from equal numbers of wild type and *Atrx* cKO muscle sections taken from three mice for each genotype. The average number of Ki67^+^ nuclei per muscle section that were counted is shown for EDL, soleus, and TA muscles along with the fraction from each that were additionally MyoD^+^ or Pax7^+^. Numbers at the top of each bar are the absolute number of Ki67^+^ nuclei that were counted for each group. (C) Ki67^+^, MyoD^+^ cells (arrowheads) were deficient for Atrx labeling in TA (shown) and EDL muscles from the *Atrx* cKO mice, whereas these cells from WT mice were positive for Atrx labeling.

To confirm that the majority of Ki67^+^ proliferating cells were Atrx-deficient myoblasts, we generated another cohort of five week-old animals for co-labeling experiments with Pax7, MyoD, and Atrx antibodies. Three different muscles (EDL, Soleus and TA) were examined for myofiber-associated Ki67^+^ cells that were also either MyoD or Pax7 positive. Equivalent numbers of tissue sections for each muscle type were examined from *Atrx* cKO and WT mice. Similar to our earlier experiments we observed an approximately three-fold increase in the absolute number of Ki67^+^ cells. More importantly, we also observed a similar increase in the number of Ki67^+^, MyoD^+^ and Ki67^+^, Pax7^+^ double-labeled cells (Fig 5A and 5B). While some Ki67^+^ cells in the muscles lacked the expression of these myoblast markers, the majority was found to be MyoD^+^ in both WT (78%) and *Atrx* cKO (73%) muscle sections (Fig 5A and 5B). Pax7 was similarly co-labeled in the majority of the Ki67^+^ nuclei, though with reduced frequency (52% in WT; 51% in Atrx cKO muscle). Co-labeling of EDL and TA muscle for Ki67, MyoD, and Atrx proteins further confirmed that the Ki67^+^ myoblasts were derived from cells that had been made deficient for Atrx (0/18 Ki67^+^,MyoD^+^ *Atrx* cKO nuclei were Atrx^+^; 9/12 Ki67^+^,MyoD^+^ WT nuclei were Atrx^+^) (Fig 5C). Additional EDL and TA sections labeled for MyoD and Atrx did not indicate the presence of any myoblasts that had escaped the *Myf5*-cre-mediated *Atrx* knockout (0/47 MyoD^+^ *Atrx* cKO nuclei were Atrx^+^; 25/31 MyoD^+^ WT nuclei were Atrx^+^) (S6 Fig). Collectively, these results confirm that the increase in muscle mass in *Atrx* cKO animals results from the prolonged expansion of the satellite cell-derived Atrx^-^ myoblasts.

## Discussion

A balance in muscle stem cell production and committed progenitor production is essential for normal muscle growth. Defects in satellite stem cell activation and the production of new myoblasts (committed progenitors) that repair damaged muscle contribute significantly to the pathology of muscular dystrophies [23, 24]. Satellite cells represent the stem cell pool that accounts for 30–35% of muscle fiber-associated nuclei in early postnatal muscle and declines to 2–7% by adulthood [25]. Importantly, they can be divided into two populations. The Pax7^+^/Myf5^-^ cells account for ~10% of the pool, grow slowly, divide asymmetrically to repopulate the stem cell niche, and are considered the satellite stem cells. Alternatively, Pax7^+^/Myf5^+^ cells proliferate rapidly, divide symmetrically, and generate committed myoblasts that differentiate and fuse to the muscle fiber [26]. Recently, it was shown that dystrophin interacts with the cell polarity machinery, and in its absence leads to a defect in asymmetric cell division that is critically required for the robust production of committed myoblasts [23]. The *mdx* dystrophic mice have normal numbers of satellite stem cells when freshly isolated, but show a 2.5-fold increase after 72 hours in culture. This increase is not observed in WT mice, but comes at the expense of committed progenitors due to an 80% reduction in asymmetric divisions. Hence, the inability to generate sufficient numbers of committed myoblasts results in poor regeneration of the muscle.

Previously, we demonstrated that mice inactivated for Atrx had normal numbers of Pax7^+^ satellite stem cells that fail to expand normally, leading to reduced muscle mass and kyphosis during development and poor recovery after injury [5]. However, for *Atrx* cKO mice, this defect arises from increased replication stress, genomic instability, and cell death of the rapidly expanding committed myoblast population, and not a defect in asymmetric division. One key difference is that these mice recover muscle mass from the continued accretion of myoblasts beyond three weeks of age. As such, we propose that satellite stem cells provide the capacity for muscle mass recovery in *Atrx* cKO mice. Elucidation of the mechanism underlying prolonged satellite cell activation and myoblast proliferation could provide therapeutic targets for conditions such as cancer cachexia, aging sarcopenia, or various muscular dystrophies.

At three weeks of age, muscle satellite cell proliferation wanes and cells become mitotically quiescent. It is believed that quiescence is mediated by changes within the stem cell niche, including reduced Wnt and Notch signaling, which have both been shown to promote/maintain the cycling status of satellite cells [27]. Other studies have suggested that extracellular matrix proteins can suppress stem cell proliferation. Fibronectin has been shown to regulate Wnt7a signaling and satellite cell expansion, while heparin sulfate proteoglycans are known to bind and sequester growth factors to suppress proliferation. The stem cell niche is also influenced by other cell types (eg. pericytes), the vasculature, and neural networks to mediate the status of the satellite cells. Indeed, this plasticity provides the ability for satellite cells to be reactivated upon muscle injury or damage. In this regard, exercise has been shown to accelerate Wnt signaling which subsequently activates satellite cells [28]. In addition, Notch signaling declines with age and this is thought to reduce the regenerative capacity in older animals.

Given the influence of the stem cell niche on satellite cell growth and quiescence, it suggests that the muscle stem cell niche of *Atrx* cKO mice may be altered such that the satellite stem cells continue to replicate beyond three weeks of age in order to compensate for reduced muscle mass. Alternatively, another study has shown that DNA double strand breaks are more efficiently repaired in satellite stem cells compared with committed progeny and that they respond to these DSBs by differentiating and showing less apoptosis [29]. This study would suggest that following the rapid expansion of muscle mass in the first three weeks following birth, the slow proliferation of the satellite stem cells are more suitably equipped to deal with DNA damage generated by Atrx loss allowing for myoblast differentiation, fusion, and continued growth of the myofiber. Indeed, our data cannot distinguish between prolonged proliferation or delayed differentiation. The genomic damage incurred by the satellite cells will lengthen the cell cycle and delay differentiation, however it seems unlikely that this will last two weeks beyond the time when myogenesis normally ceases. If the satellite cell niche switches to a quiescent microenvironment at 3 weeks, then one would expect that at 5 weeks the number of BrdU or Ki67 positive cells would greatly diminish, similar to what is observed in WT cells. The continued incorporation of BrdU and positive labeling for Ki67 would argue that the cells remain proliferative. Moreover, we did not observe a reduction in satellite stem cells, which would be expected if a large proportion of the satellite stem cells contained significant DSBs that led to their subsequent differentiation. While we favour prolonged proliferation it remains likely that both mechanisms function to contribute to the growth of the muscle beyond three weeks of age.

Our observations support the ideas that there is an intrinsic requirement for fiber size that must be met before quiescence is fully achieved, and that a minimum number of nuclei are required to support a larger cytoplasmic volume within the fiber syncytium. In the context of the α-thalassemia mental retardation syndrome, our results suggest that a delay in reaching developmental motor milestones, such as time to sit and walk, may be a direct result from delayed muscle growth [30]. Similarly, any improvements in motor function may at least in part be explained by this prolonged period of developmental muscle growth, particularly since there is no continued degeneration of the muscle like there is in muscular dystrophies. Indeed, this is in sharp contrast to DMD patients and *mdx* mice who also experience persistent damage to mature myofibers caused by the loss of dystrophin and the dystroglycan complex that compromises membrane integrity and requires constant repair. This degeneration/regeneration cycle is further compromised by dystrophin loss in satellite cells that is required to facilitate asymmetric division, committed myoblast expansion and repair [23]. Further investigation on the composition of the satellite stem cell niche during periods of inactivity, disease, or delayed developmental growth should help tease apart the intrinsic and extrinsic mechanisms regulating muscle size.

## Supporting information

S1 Fig ARRIVE guidelines checklist. (PDF)

S2 Fig Hind limb muscle weights from five week-old *Atrx* cKO and WT mice. (A) TA, EDL, and soleus muscles were all significantly smaller in the *Atrx* cKO mice measured at five weeks (mean ± SEM; ***p<0.001 by student’s t-test, WT n = 18, cKO n = 20). (B) When normalized to body weight, the *Atrx* cKO TA and EDL muscles were still significantly smaller, though the difference was marginal. There was no difference in the normalized weights of the soleus muscles (mean ± SEM; *p<0.05).
(TIFF)

S3 Fig Gross morphology and fiber type composition in *Atrx* cKO muscle. TA and EDL muscle sections from 5 week-old mice were labeled for laminin (green) and either myosin heavy chain (MHC) type IIb (red) or MHC type IIa + type I (red). No consistent differences in the appearance of the muscle fibers or MHC labeling was noted. Muscles from three mice/group were analyzed.
(TIFF)

S4 Fig **qPCR expression analyses in oxidative soleus muscle** (A) and glycolytic EDL (B) muscle of five week-old mice. We examined the expression of the negative regulators of muscle hypertrophy *myostatin* and *Inhibin A*, and their receptor *Acvr2A*; the pro-hypertrophic *IL-6*, myomaker, and follistatin; and *TNF*, which is associated with both muscle wasting and with the promotion of myoblast proliferation. Relative fold change was calculated for individual *Atrx* cKO animals (n = 3) with respect to littermate controls (n = 3). Error bars indicate the range, as determined using the standard deviation of the ΔCt values. Asterisk indicates a statistical difference (p<0.05) between *Atrx* cKO and WT sample expression, determined using a Student’s t-test to compare target Ct values normalized to their respective reference (GAPDH) Ct values.
(TIFF)

S5 Fig Distribution of myofiber length in WT and Atrx cKO mice. (A) Distribution of individual myofibers from the EDL muscle as plotted by myofiber length in *Atrx* cKO and littermate controls. Median and quartile points are represented for each animal group by red and black hash marks, respectively (3 week WT, cKO: n = 111, 122 fibers; 5 week WT, cKO: n = 170, 178 fibers; adult WT, cKO: n = 186, 214 fibers). (B) Average length per fiber in EDL muscle in *Atrx* cKO and littermate controls (mean ± SEM; ***p<0.001 by student’s t-test).
(TIFF)

S6 Fig Atrx labeling in MyoD^+^ nuclei within *Atrx* cKO muscle was completely lacking. MyoD^+^ nuclei from *Atrx* cKO TA and EDL muscle displayed no Atrx labeling (0/47 nuclei), whereas the majority of WT MyoD^+^ nuclei were Atrx^+^ (25/31 nuclei). The open arrows in the top panels indicate interstitial nuclei in the *Atrx* cKO TA muscle which were Atrx^+^; all other arrows indicate myofiber-associated MyoD^+^ cells. Including sections additionally labelled for Ki67 (Fig 5C), 100% of the MyoD^+^ nuclei (0/65) in the *Atrx* cKO muscle displayed no labelling for Atrx above background; 79% of the MyoD^+^ nuclei (34/43) in WT muscle did display Atrx protein expression.
(TIFF)
