# Supplementary figures and images for: Recovery from impaired muscle growth arises from prolonged postnatal accretion of myonuclei in Atrx mutant mice
Source: PLoS One. 2017 Nov 2;12(11):e0186989. doi: 10.1371/journal.pone.0186989 (PMC5667798; doi:10.1371/journal.pone.0186989)

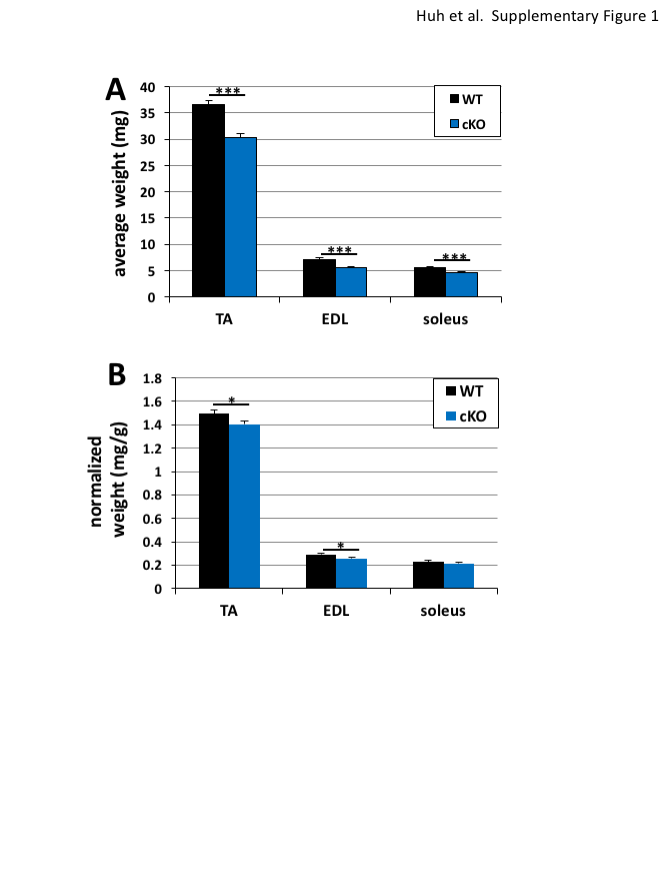

Supplement: S2 Fig — (A) TA, EDL, and soleus muscles were all significantly smaller in the Atrx cKO mice measured at five weeks (mean ± SEM; ***p<0.001 by student’s t-test, WT n = 18, cKO n = 20). (B) When normalized to body weight, the Atrx cKO TA and EDL muscles were still significantly smaller, though the difference was marginal. There was no difference in the normalized weights of the soleus muscles (mean ± SEM; *p<0.05). (TIFF) [file pone.0186989.s002.tiff]

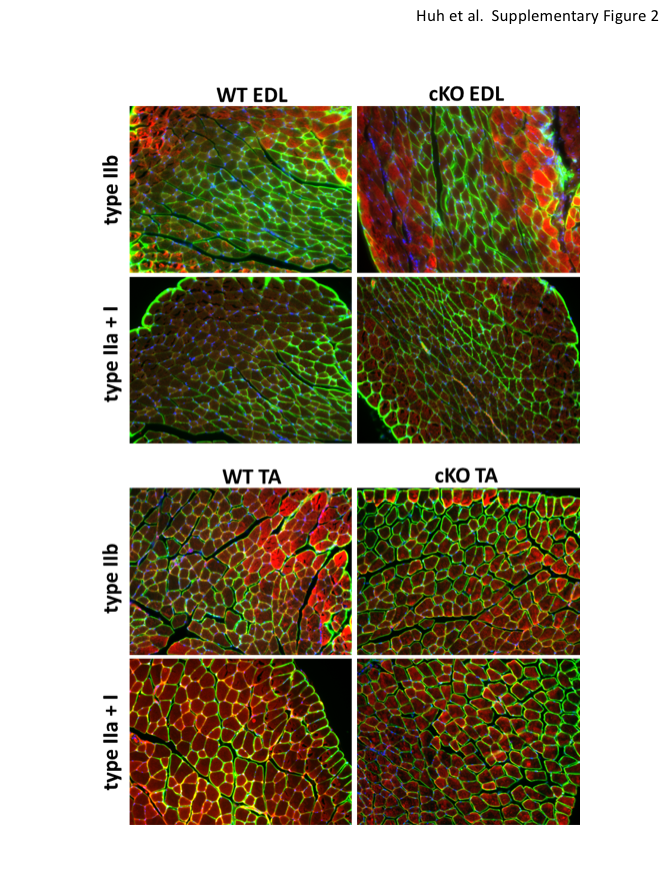

Supplement: S3 Fig — TA and EDL muscle sections from 5 week-old mice were labeled for laminin (green) and either myosin heavy chain (MHC) type IIb (red) or MHC type IIa + type I (red). No consistent differences in the appearance of the muscle fibers or MHC labeling was noted. Muscles from three mice/group were analyzed. (TIFF) [file pone.0186989.s003.tiff]

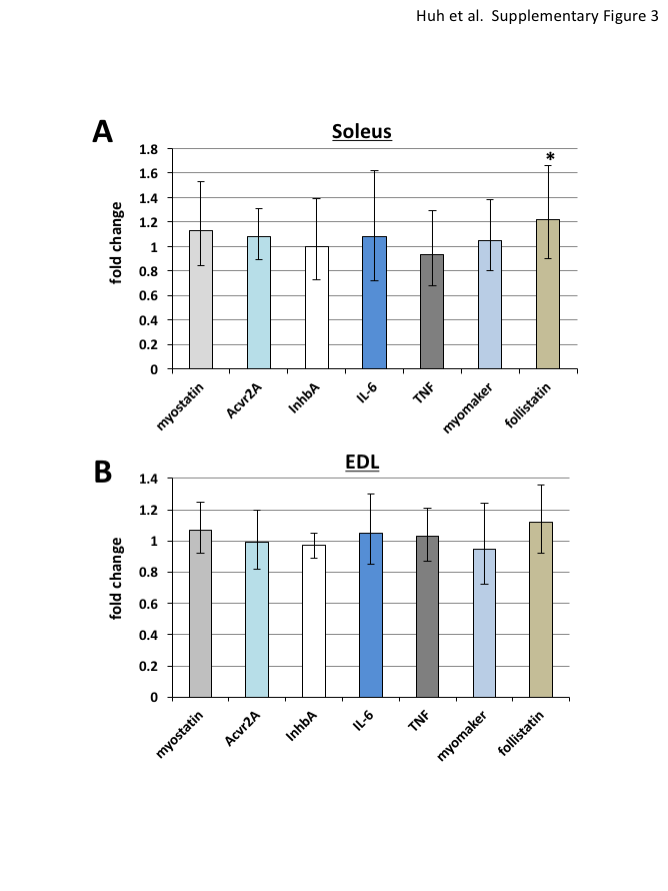

Supplement: S4 Fig — qPCR expression analyses in oxidative soleus muscle (A) and glycolytic EDL (B) muscle of five week-old mice. We examined the expression of the negative regulators of muscle hypertrophy myostatin and Inhibin A, and their receptor Acvr2A; the pro-hypertrophic IL-6, myomaker, and follistatin; and TNF, which is associated with both muscle wasting and with the promotion of myoblast proliferation. Relative fold change was calculated for individual Atrx cKO animals (n = 3) with respect to littermate controls (n = 3). Error bars indicate the range, as determined using the standard deviation of the ΔCt values. Asterisk indicates a statistical difference (p<0.05) between Atrx cKO and WT sample expression, determined using a Student’s t-test to compare target Ct values normalized to their respective reference (GAPDH) Ct values. (TIFF) [file pone.0186989.s004.tiff]

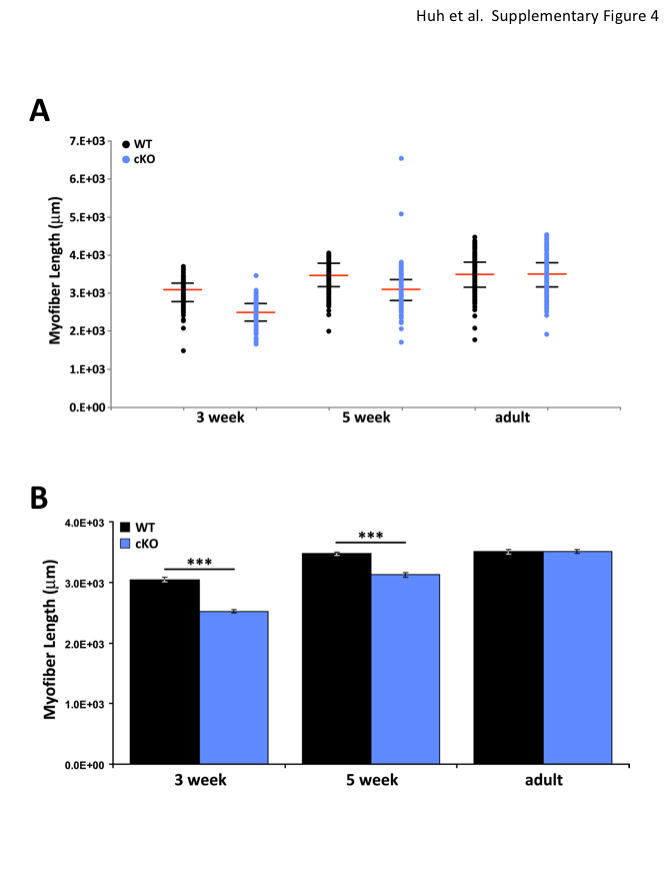

Supplement: S5 Fig — (A) Distribution of individual myofibers from the EDL muscle as plotted by myofiber length in Atrx cKO and littermate controls. Median and quartile points are represented for each animal group by red and black hash marks, respectively (3 week WT, cKO: n = 111, 122 fibers; 5 week WT, cKO: n = 170, 178 fibers; adult WT, cKO: n = 186, 214 fibers). (B) Average length per fiber in EDL muscle in Atrx cKO and littermate controls (mean ± SEM; ***p<0.001 by student’s t-test). (TIFF) [file pone.0186989.s005.tiff]

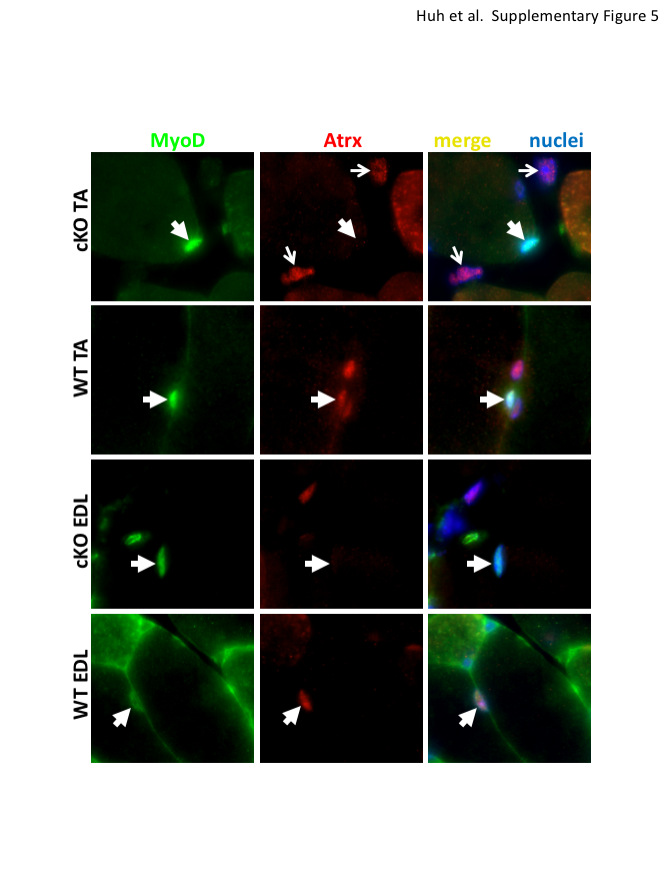

Supplement: S6 Fig — MyoD+ nuclei from Atrx cKO TA and EDL muscle displayed no Atrx labeling (0/47 nuclei), whereas the majority of WT MyoD+ nuclei were Atrx+ (25/31 nuclei). The open arrows in the top panels indicate interstitial nuclei in the Atrx cKO TA muscle which were Atrx+; all other arrows indicate myofiber-associated MyoD+ cells. Including sections additionally labelled for Ki67 (Fig 5C), 100% of the MyoD+ nuclei (0/65) in the Atrx cKO muscle displayed no labelling for Atrx above background; 79% of the MyoD+ nuclei (34/43) in WT muscle did display Atrx protein expression. (TIFF) [file pone.0186989.s006.tiff]
